# Diagnosis and Treatment of Abdominal Arterial Bleeding After Radical Gastrectomy: a Retrospective Analysis of 1875 Consecutive Resections for Gastric Cancer

**DOI:** 10.1007/s11605-015-3049-z

**Published:** 2015-12-14

**Authors:** Jie Yang, Xin-hua Zhang, Yong-hui Huang, Bin Chen, Jian-bo Xu, Chuang-qi Chen, Shi-rong Cai, Wen-hua Zhan, Yu-long He, Jin-ping Ma

**Affiliations:** Department of Gastrointestinal Surgery, The First Affiliated Hospital of Sun Yat-sen University, 510080 No. 58, 2nd Zhongshan Road, Guangzhou, Guangdong Province China; Department of Interventional Radiology, The First Affiliated Hospital of Sun Yat-sen University, Guangzhou, Guangdong Province China

**Keywords:** Gastric cancer, Radical gastrectomy, Lymphadenectomy, Postoperative arterial bleeding, Hemostasis, Angiography, Re-laparotomy

## Abstract

**Background:**

Massive abdominal arterial bleeding is an uncommon yet life-threatening complication of radical gastrectomy. The exact incidence and standardized management of this lethal morbidity are not known.

**Methods:**

Between January 2003 and December 2013, data from 1875 patients undergoing radical gastrectomy with D2 or D2 plus lymphadenectomy were recorded in a prospectively designed database from a single institute. The clinical data and management of both early (within 24 h) and late (beyond 24 h) postoperative abdominal arterial hemorrhages were explored. For late bleeding patients, transcatheter arterial embolization (TAE) and re-laparotomy were compared to determine the better initial treatment option.

**Results:**

The overall prevalence of postoperative abdominal arterial bleeding was 1.92 % (*n* = 36), and related mortality was 33.3 % (*n* = 12). Early and late postoperative bleedings were found in 6 and 30 patients, respectively. The onset of massive arterial bleeding occurred on average postoperative day 19. The common hepatic artery and its branches were the most common bleeding source (13/36; 36.1 %). All the early bleeding patients were treated with immediate re-laparotomy. For late bleeding, patients from the TAE group had a significantly lower mortality rate than that of the patients from the surgery group (7.69 vs. 56.25 %, respectively, *P* = 0.008) as well as a shorter procedure time for bleeding control (2.3 ± 1.1 vs. 4.8 ± 1.7 h, respectively, *P* < 0.001). Four rescue reoperations were performed for TAE failures; the salvage rate was 50 % (2/4). Ten patients developed massive re-bleeding after initial successful hemostasis by either TAE (5/13) or open surgery (5/16). Three out of the 10 re-bleeding patients died of disseminated intravascular coagulation (DIC), while the other 7 recovered eventually by repeated TAE and/or surgery.

**Conclusion:**

Abdominal arterial bleeding following radical gastrectomy tends to occur during the later phase after surgery, with further complications such as abdominal infection and fistula(s). For late bleeding, TAE can be considered as the first-line treatment when possible.

**Electronic supplementary material:**

The online version of this article (doi:10.1007/s11605-015-3049-z) contains supplementary material, which is available to authorized users.

## Introduction

Although mortality and morbidity of gastric cancer surgery have decreased in recent decades, especially in high-volume centers,^1^–^3^ postoperative massive hemorrhage is still a devastating complication with a high mortality rate that ranges from 2.6 to 26.6 %.^4^–^8^ Among frequent causes, abdominal arterial bleeding, mainly from the branches of the celiac trunk or the common hepatic artery, is a specific postoperative morbid event after D2 or D2 plus lymphadenectomy. Gastrectomy with D2 or D2 plus lymph node dissection has been the long-standing standard procedure for locally advanced gastric cancer in most hospitals in Eastern Asia.

Postoperatively, early arterial bleeding is reported to have a more favorable prognosis than late bleeding and immediate re-laparotomy is considered the mainstay of treatment. However, the morbidity and mortality of emergency re-laparotomy are high in late postoperative period.^3^,^9^ With the advances in radiology techniques, angiography and transcatheter arterial embolization (TAE), as an alternative to re-operation, have been widely used for the diagnosis and treatment of postoperative arterial bleeding from pancreatic or other abdominal surgeries.^10^–^13^

There have been numerous reports regarding postoperative arterial bleeding after pancreatic resections, but very few studies are done on postoperative arterial bleeding after gastric cancer surgery. The clinical features of postoperative arterial bleeding in patients who underwent radical gastrectomy remain unclear. Additionally, the most appropriate diagnostic and therapeutic protocol is still a matter of controversy.

In this study, the clinical features, management, and outcome of patients who experienced abdominal arterial bleeding following radical gastrectomy were analyzed over an 11-year period.

## Materials and Methods

### Patient Selection

This is a retrospective study performed in a single high-volume gastric cancer center. From January 2003 to December 2013, 1875 consecutive gastric cancer patients who underwent D2 or D2 plus gastrectomy were analyzed from our prospectively designed database. Only confirmed abdominal arterial bleeding cases after radical gastrectomy, with systematic lymphadenectomy, were included in the analysis. Patients who underwent radical gastrectomy with standard D2 or D2 plus lymph node dissection with curative intent for gastric cancer and had a confirmed diagnosis of abdominal arterial hemorrhage, either by transcatheter arterial angiography or open surgery, were included in the study. Patients who underwent laparoscopic surgery, palliative surgery, reduction surgery, bypass surgery, or biopsy alone and those who suffered transient hemorrhage, hemorrhage from anastomotic line, non-arterial hemorrhage (i.e., venous hemorrhage), diffuse gastritis, preexisting coagulation disorders, or other unclear sources of hemorrhage were excluded from the study.

Of note, in our series, anticoagulation therapy or antiplatelet therapy was not used perioperatively. Neoadjuvant chemotherapies were not performed in all patients. Stages of gastric cancer were classified using the seventh edition of UICC/AJCC TNM stage system.

### Ethics Statement

Ethical subcommittee of The First Affiliated Hospital of Sun Yat-sen University approved this retrospective study (approval number: 20140429). Written consent was obtained from the patients for their information to be stored in the hospital database and used for research.

### Radical Gastrectomy with Systematic Lymphadenectomy

All included patients underwent total or subtotal gastrectomy with D2 or greater extended lymph node dissection, based on the recommendation from the Japanese Research Society for Gastric Carcinoma, with curative intent.^22^ All operations were done by experienced surgeons who had performed at least 50 D2 radical gastrectomies, which required the systematic dissection of lymph nodes in the first tier (perigastric) and the second tier (along the celiac artery and its branches). Based on preoperative staging, D2 plus or D3 lymphadenectomy was performed on selected patients. The types of gastrectomy were selected largely based on the size and location of the primary tumors. Omentobursectomy was performed for tumors located on the posterior gastric wall, especially those penetrating the serosa. Combined organ resections, such as splenectomy and distal pancreatectomy, were carried out if the primary tumor directly invaded the adjacent organs to obtain an R0 resection. Reconstruction types included Billroth type I/II and Roux-en-Y esophagojejunostomies. After anastomosis, hemostasis at the anastomotic line was checked routinely. Warm distilled water lavage and soaking were routinely performed for inactivation of deciduous gastric cancer cells and to find potential hemorrhagic spots. For all patients, one silicone drain was placed around the anastomotic site through the Winslow foramen before closure. Another drain was added at the splenic fossa for patients with total gastrectomy, especially with combined pancreatectomy or splenectomy.

### Definition of Early and Late Bleeding

Due to a lack of generally accepted definition and classification of postoperative bleeding for gastric cancer, we referenced and adopted the classification of postoperative hemorrhage for pancreatic surgery.^14^–^19^ Based on the onset time of hemorrhage, patients who suffered from arterial bleeding within and beyond 24 h after surgery were assigned to an early arterial bleeding group and a late arterial bleeding group, respectively. For the late bleeding group, based on the primary interventional measure, we divided patients into the TAE first group or re-laparotomy first group. Sentinel bleeding was defined as any kind of minor bleeding that required no intervention and often preceded one or more major bleeding events.^20^,^21^ Re-bleeding was defined as recurrent active bleeding that required further intervention after the initial hemostatic procedure.

### Diagnosis and Treatment of Abdominal Arterial Bleeding

Abdominal arterial bleeding following gastrectomy was alerted by series of clinical manifestations including massive warm and bright red bleeding from an abdominal drain, tachycardia, hemodynamic instability, sudden abdominal pain, distension, etc. Hemodynamic instability was defined as a quick decease in the mean arterial pressure (MAP <65 mmHg) prior to fluid resuscitation or blood transfusion. Fluid resuscitation was initiated prior to and during emergency interventions. The following diagnostic procedures were considered: bedside ultrasonography, abdominocentesis, or angiography. A decision to proceed with TAE or re-laparotomy was based on hemodynamic status, extent of bleeding, risk factor, onset time of bleeding, etc.

### Radiological Intervention

**Fig. 1:**
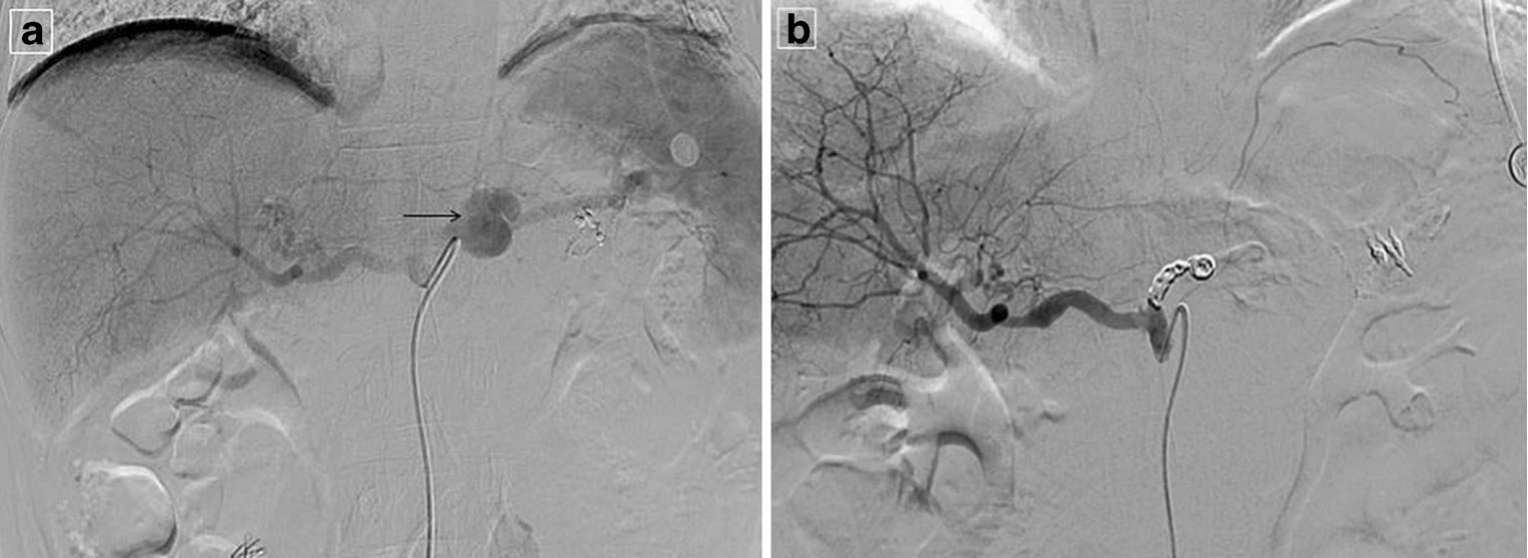
Bleeding pseudoaneurysm from the splenic artery. A 60-year-old man with advanced gastric cancer presented with massive hematemesis 18 days after radical gastrectomy. **a** Celiac angiogram demonstrates a large pseudoaneurysm (*arrow*) arising at the proximal of the splenic artery. **b** The splenic artery was successfully embolized proximally to pseudoaneurysm with coils

**Fig. 2:**
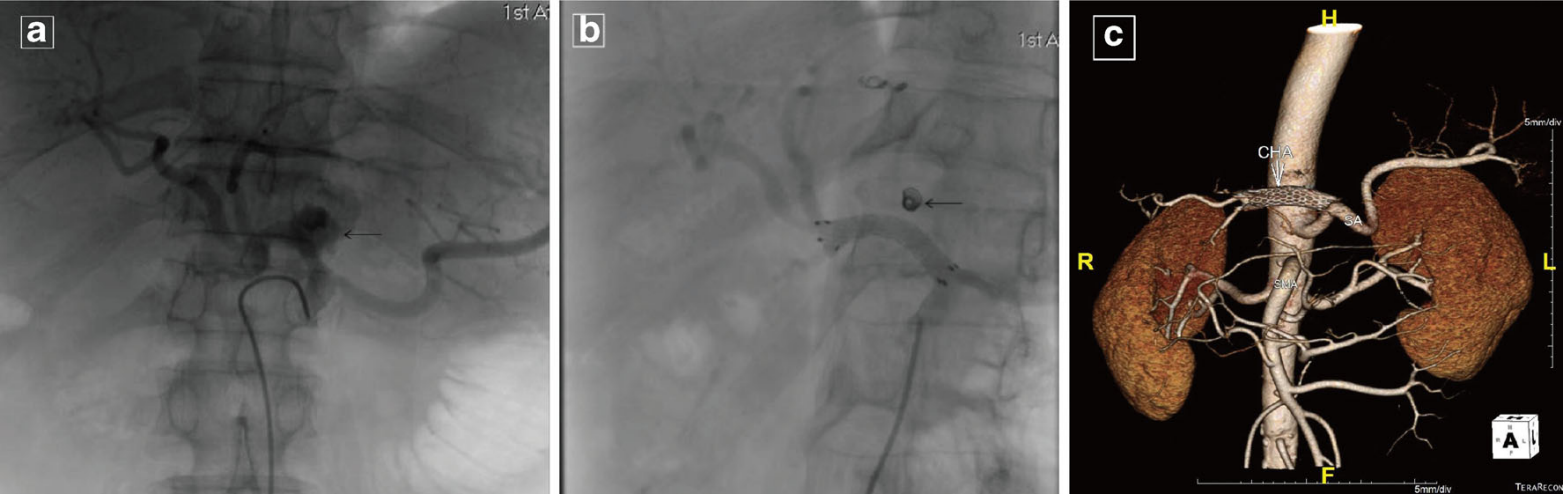
Bleeding pseudoaneurysm from the common hepatic artery. A 54-year-old man with distal gastrectomy for gastric cancer suddenly presented with severe abdominal pain at home, with a quick drop of hemoglobin 37 days after surgery. **a** Angiogram of the celiac axis shows a bleeding pseudoaneurysm (*arrow*) originating from the common hepatic artery. **b** The pseudoaneurysm was successfully planted with covered stent graft. The *arrow* shows the failed placing embolized coils. **c** The CT image reconstruction of abdominal arteries; *arrow* shows the covered stent graft

In our hospital, a team of interventional radiologists was able to respond within 10 min, 24 h per day. All angiography and TAE were performed under local anesthesia. Celiac and superior mesenteric arterial angiographies were routinely performed to detect the bleeding in all cases. Positive angiographic findings included extravasations of contrast medium, pseudoaneurysms, or fusiform aneurysms. When the bleeding source was demonstrated at angiography, embolization was performed as selectively as possible. The choices between embolization and covered stent graft depended on the discretion of interventional radiologists and anatomic conditions. Embolization coils were placed from the distal part to the proximal part of the bleeding point to prevent retrograde perfusion through collateral vessels (Figs. 1 and 2, Supporting Files 1 and 2). A re-laparotomy was performed as a rescue treatment for a negative finding or failure of radiological intervention. Liver function was closely monitored after the procedure. Abdominal CT scans were performed to check for fluid collections after successful bleeding control by TAE.

### Surgical Re-exploration

All emergency re-laparotomies were performed under general anesthesia. In cases with less than 14 postoperative days, bleeding vessels could be generally detected and confirmed. However, in cases with severe vascular damage, extensive adhesion due to unconfined anastomotic leakage, or more than 2 weeks after gastrectomy, bleeding vessels could not always be confirmed. Surgical procedures ranged from simple ligation of bleeding arteries, over sewing the bleeding site, to vascular reconstruction, and to bleeding organ resection, like splenectomy. Gauze packing was used as a salvage measure when bleeding control failed.

### Definitions and General Management of Relevant Complications

Intra-abdominal infection was confirmed by a positive culture of the intra-abdominal fluid collected from patients who had a body temperature higher than 38 °C. Local sepsis and intra-abdominal abscess were defined as direct drainage of abscess from the peritoneal cavity. Pancreatic fistula was defined when drain fluid is >20 ml/24 h, after 3 days postoperatively with amylase activity exceeding three times that of the serum. Anastomotic leakage was diagnosed by digestive tract radiography. Ultrasound-guided additional drains were usually placed if intra-abdominal fluid collection was found.

### Statistical Analysis

All statistical analyses were performed using the Statistical Package for Social Science (SPSS) version 19.0 for Windows (IBM, Chicago, IL, USA). A Student’s *t* test and a Mann-Whitney *U* test were used to compare continuous variables. Fisher’s exact test was used to compare categorical variables. A *P* value of <0.05 was considered to indicate statistical significance.

## Results

### Overall Incidence

In 1875 patients with radical gastrectomy, 22 patients who experienced transient hemorrhage, anastomotic hemorrhage, venous hemorrhage, preexisting coagulation disorders, and unclear sources of massive postoperative hemorrhage were excluded. A total of 36 abdominal arterial bleeding cases were confirmed. The overall prevalence of postoperative abdominal arterial hemorrhage was 1.92 % (31 men and 5 women; age 59.9 ± 8.0 years). The demographic and clinical data are summarized in Table 1.

**Table 1:** Demographic and clinical presentation of arterial bleeding after radical gastrectomy

Characteristics	Total (*n* = 36)	Early (*n* = 6)	Late (*n* = 30)	*P* value^a^
Age (years; mean ± SD)	59.9 ± 8.0	63.7 ± 7.5	59.0 ± 8.0	0.297
Sex, *n* (%)				
Male	31 (86.1)	5 (83.3)	26 (86.7)	1.000
Female	5 (13.8)	1 (16.7)	4 (13.3)	
Concomitant disease, *n* (%)				
ASA I + II	27 (75.0)	4 (66.7)	23 (76.7)	0.627
ASA III	9 (25.0)	2 (33.3)	7 (23.3)	
pTNM stage, *n* (%)				
I + II	16 (44.4)	5 (83.3)	11 (36.7)	0.069
III + IV	20 (55.5)	1 (16.7)	19 (63.3)	
Type of gastrectomy, *n* (%)				
Total gastrectomy	20 (55.5)	3 (50.0)	17 (56.7)	1.000
Subtotal gastrectomy	16 (44.4)	3 (50.0)	13 (43.3)	
Extent of lymphadenectomy, *n* (%)				
D2	24 (66.7)	6 (100)	18 (60.0)	0.079
D2 plus or combined organ resection	12 (33.3)	0	12 (40.0)	
Clinical presentation				
Bleeding from abdominal drain, *n* (%)	29 (80.6)	6 (100)	23 (76.7)	0.317
Gastrointestinal tract bleeding, *n* (%)	13 (36.1)	0	13 (43.3)	0.068
Severe upper abdominal pain, *n* (%)	16 (44.4)	1 (16.7)	15 (50.0)	0.196
Sentinel bleedings, *n* (%)	21 (58.3)	0	21 (70.0)	<0.001
IAI or PF or AL before bleeding, *n* (%)	24 (66.7)	0	24 (80.0)	<0.001
Hemodynamic instability, *n* (%)	25 (69.4)	5 (88.3)	20 (66.7)	0.634
Drop of Hgb (g/l; mean ± SD)	53.4 ± 17.3	49.2 ± 20.1	54.2 ± 16.9	0.640

*SD* standard deviation, *ASA* American Society of Anesthesiologists, *AL* anastomotic leakage, *PF* pancreatic fistula, *IAI* intra-abdominal infection, *Hgb* hemoglobin

^a^Early versus late

### Time of Bleeding Onset

**Fig. 3:**
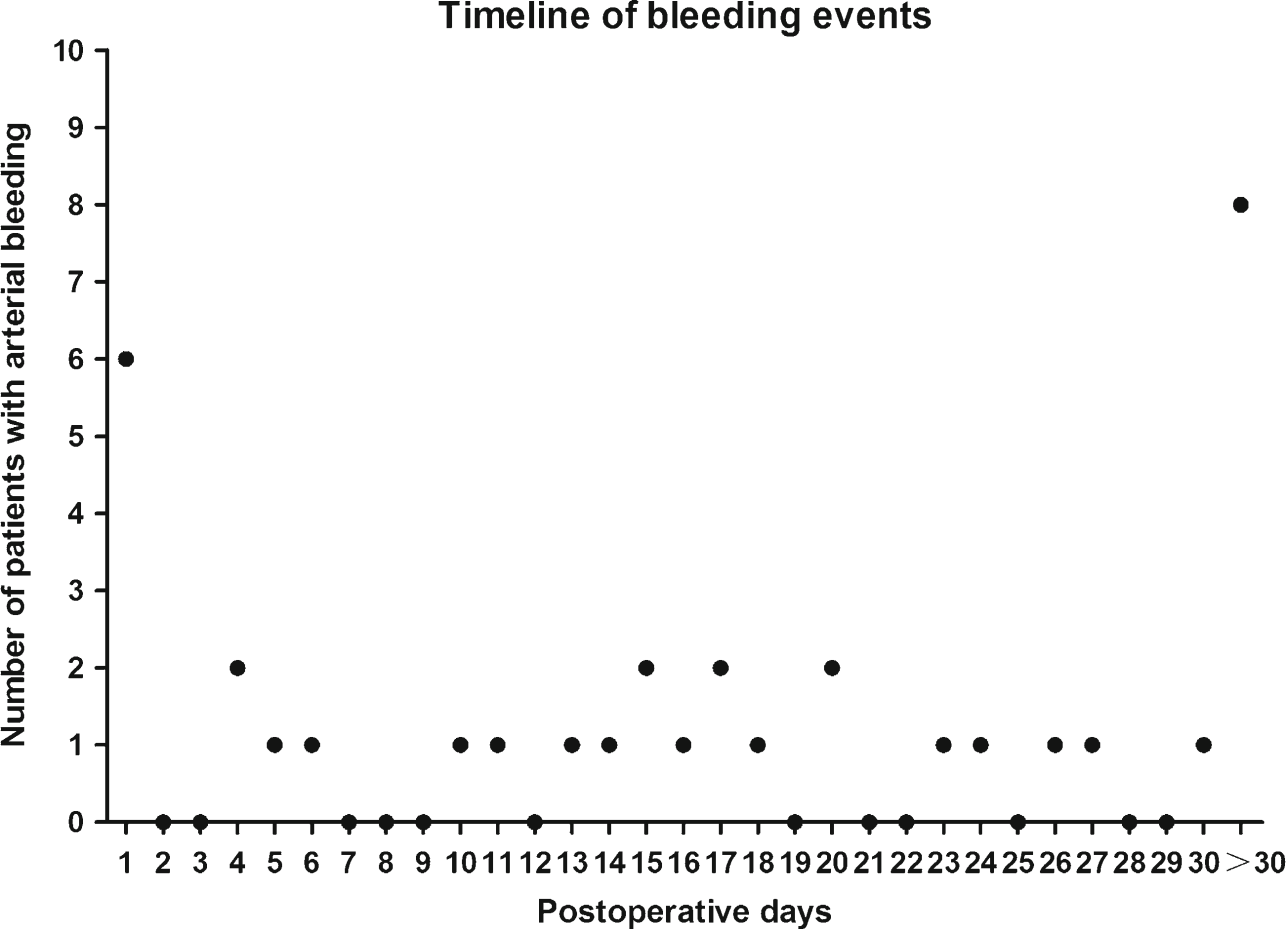
The bleeding event. The arterial bleeding events occurred 0–90 days after the surgery, with a mean of 19 days. The onset of bleeding events had a peak on day 1 and scattered from day 4 to months

The arterial hemorrhagic episodes occurred between 0 and 90 days after the surgery, with an average of 19 days (see Fig. 3 and Supplemented Table). Bleeding occurred within 24 h following gastrectomy in 6 patients (16.7 %) and over 24 h in the remaining 30 patients (83.3 %).

### Bleeding Sites

After angiography or surgical re-exploration, the primary bleeding sources were detected in the common hepatic artery (CHA) and its branches in 13 cases (13/36; 36.1 %), the splenic artery (SPA) and its branches in 10 cases, the peripancreatic arteries in 5 cases, other abdominal arteries in 6 cases, and the undetermined origin but considered as sources from abdominal arteries in 2 cases. More details of these arterial bleeding cases are shown in the Supplemented Table.

### Clinical Presentations

As the clinical characteristics and management of early and late hemorrhage were distinctive, we summarized them separately (see Tables 1 and 2).

**Table 2:** Treatment and outcome of arterial bleeding after radical gastrectomy

Variables	Total (*n* = 36)	Early (*n* = 6)	Late (*n* = 30)	*P* value^a^
Confirmative diagnostic tools, *n* (%)				
Arteriography	13 (36.1)	0	13 (43.3)	
Clinical presentations and decision	23 (63.8)	6	17 (56.7)	
Treatment for initial hemostasis, *n* (%)				
Surgery	22 (66.1)	6	16 (53.3)	
Angiography and TAE	14 (38.8)	0	14 (46.7)	
Outcome				
Mortality, *n* (%)	12 (33.3)	1 (16.7)	11 (36.7)	0.640
24-h mortality, *n* (%)	7 (19.4)	1 (16.7)	6 (16.7)	1.000
Re-bleeding, *n* (%)	10 (27.8)	1 (16.7)	9 (30.0)	0.655
24-h re-bleeding, *n* (%)	5 (13.9)	1 (16.7)	4 (13.3)	1.000
Requiring ICU stay, *n* (%)	26 (72.2)	4 (66.7)	22 (73.3)	1.000
Total RBC transfusion (*U*; median (range)	22.5 (4–68)	20 (6–30)	25.5 (4–68)	0.223
Total plasma transfusion (ml; median (range)	1600 (600–7800)	1000 (600–3200)	1900 (600–7800)	0.06

*TAE* transcatheter arterial embolization,* RBC* red blood cell, *ICU* intensive care unit

^a^Early versus late

### Early Postoperative Abdominal Arterial Bleeding

All the early postoperative bleedings were evidenced by massive increasing sanguineous fluid from the abdominal drains. No one developed an infection before bleeding. Diagnosis and bleeding sources of all 6 patients were confirmed by immediately following up with a re-laparotomy. Intraoperative insufficient hemostasis and technical mishaps were considered the primary causes of early arterial bleeding.

### Late Postoperative Abdominal Arterial Bleeding

Clinical presentations of late postoperative bleeding were varied and insidious. Bleeding was observed from surgical drains in only 17 of 30 patients (56.7 %), gastrointestinal tract in 7 patients (23.3 %), and from both in 6 patients (20 %). In late arterial bleeding, 21 out of 30 patients (70 %) presented sentinel bleeding one or more times while 24 cases (80 %) were complicated with intra-abdominal infection caused by anastomotic leakage or pancreatic fistula before bleeding.

### Treatment and Outcome

All 6 early bleeding patients were treated with immediate re-laparotomy. Of the 30 patients suffering from late bleeding, 13 were transferred to receive radiological interventions with TAE and 16 received re-laparotomy as the first treatment choice. One patient did not have the chance to receive TAE or rescue surgery due to acute hemodynamic collapse within 1 h after a dubious negative finding from angiography. The overall mortality rate of postoperative arterial bleeding was 33.3 % (*n* = 12). One was from early bleeding (1/6, 16.7 %), and the others were from late bleeding (11/30, 36.7 %). In detail, 2 patients died of acute hemodynamic collapse directly, 3 as a result of hemostatic failure after disseminated intravascular coagulation (DIC), and 7 due to multiple organ dysfunction syndrome (MODS) despite initial successful hemostasis. Although the mortality rate in late bleeding was higher than that in early bleeding, the difference was not statistically significant (36.7 vs. 16.7 %, respectively, *P* = 0.640).

### TAE Versus Surgery in Late Bleeding Patients

Safety and efficacy between TAE and re-laparotomy as the primary treatment option were compared in the late bleeding cases. Despite the TAE first group having a late bleeding onset time, there were no other significant differences between the two groups in regard to demographics and clinical characteristics (see Table 3). From our series, the TAE first group had a significantly lower mortality rate than the surgery first group (7.69 vs. 56.25 %, respectively, *P* = 0.008) where 9 patients out of 16 died. One died during the surgery, 1 died of re-bleeding and DIC within 24 h after re-laparotomy, and 7 died of MODS despite bleeding control achieved. In the TAE group, only 1 patient died of recurrent massive bleeding with DIC. A significantly shorter procedure time was recorded for hemostasis in the TAE group than that in the surgery group (2.3 ± 1.1 vs. 4.8 ± 1.7 h, respectively, *P* < 0.001). The ICU requirement rate, total RBC, and plasma transfusion volume were higher in the surgery group, but the differences were not statistically significant.

**Table 3:** Demographics, clinical characteristics, and outcome between TAE and surgery groups

Variables	Surgery (*N* = 16)	TAE (*N* = 13)	*P* value
Age (years; mean ± SD)	61.31 ± 8.50	57.69 ± 5.99	0.191
Sex, *n* (%)			
Male	12 (75)	13 (100)	0.107
Female	4 (25)	0	
Concomitant disease, *n* (%)			
ASA I or II	13 (81.3)	9 (69.2)	0.667
ASA III	3 (18.7)	4 (30.8)	
pTNM stage, *n* (%)			
I or II	7 (37.5)	3 (23.1)	0.433
III or IV	9 (62.5)	10 (76.9)	
Type of gastrectomy, *n* (%)			
Total gastrectomy	10 (62.5)	7 (53.8)	0.638
Subtotal gastrectomy	6 (37.5)	6 (46.2)	
Extent of lymphadenectomy, *n* (%)			
D2	8 (50)	9 (69.2)	0.451
D2 plus or combined organ resection	8 (50)	4 (30.8)	
Bleeding site			
Common hepatic artery and its branches, *n* (%)	7 (43.8)	6 (46.2)	1.000
Splenic artery and its branches, *n* (%)	5 (31.3)	4 (30.8)	1.000
Onset of first major bleeding (POD, days; mean ± SD)	15.9 ± 9.7	32.1 ± 18.5	<0.001
Hemodynamic instability when bleeding, *n* (%)	12 (75.0)	7 (53.8)	0.207
Drop of Hgb (g/l; mean ± SD)	53.7 ± 17.7	54.0 ± 17.1	0.843
Sentinel bleedings, *n* (%)	11 (68.7)	10 (76.9)	0.697
Intra-abdominal infection before bleeding, *n* (%)	13 (81.3)	11 (84.6)	1.000
Anastomotic leakage before bleeding, *n* (%)	2 (12.5)	5 (38.4)	0.192
Pancreatic fistula before bleeding, *n* (%)	2 (12.5)	4 (30.8)	0.364
Time of hemostasis^a^ (h; mean ± SD)	4.8 ± 1.7	2.3 ± 1.1	<0.001
Outcome			
Mortality rate, *n* (%)	9 (56.3)	1 (7.7)	0.008
24-h mortality rate, *n* (%)	4 (25.0)	1 (7.7)	0.343
Re-bleeding, *n* (%)	4 (25.0)	5 (38.5)	0.688
24-h re-bleeding, *n* (%)	1 (6.25)	3 (23.1)	0.299
Requiring ICU stay, *n* (%)	14 (87.5)	7 (53.8)	0.092
MODS, *n* (%)	7 (43.8)	0	0.008
DIC, *n* (%)	1 (6.3)	1 (7.7)	1.000
Total RBC transfusion (*U*, median (range)	30 (10–68)	20 (10–62)	0.496
Total plasma transfusion (ml, median (range)	1900 (800–7400)	2150 (600–7800)	0.702

*TAE* transcatheter arterial embolization, *DIC* disseminated intravascular coagulation, *MODS* multiple organ dysfunction syndrome, *POD* postoperative day, *HGB* hemoglobin

^a^represents the time of actual a OR/procedure time

Ten patients developed massive re-bleeding after initial successful hemostasis by either TAEs (5/13) or open surgeries (5/16). The re-bleeding rate in the TAE group was higher than that in the surgery group, but not statistically significant (38.46 vs. 25.0 %, respectively, *P* = 0.688). Three out of the 10 re-bleeding patients died of DIC, while the other 7 recovered eventually by repeated TAEs and/or surgeries. Further information and management of re-bleeding are shown in Table 4.

**Table 4:** Information and managements of re-bleeding cases

Patient no./sex/age (years)	Initial bleeding			Recurrent bleeding			
	Onset	Initial treatment	Bleeding site	Onset (POD, days)	Treatment	Bleeding site	Outcome
13/M/56	31	Surgery	SPA	34	Surgery	PHA	Recovered
23/M/60	5	Surgery	Marginal artery of the transverse colon	15	Surgery after TAE failed	CHA	Recovered
22/F/69	10	Surgery	Upper pole of the spleen	53	TAE	Stump of the SPA	Recovered
24/M/65	36	TAE	LHA	37	TAE	PHA	Recovered
28/M/48	24	TAE	CHA	30	TAE	Accessory hepatic artery originated from the SMA	Recovered
31/M/58	90	TAE	Gastroduodenal artery	90	TAE	PHA	Recovered
32/M/65	26	TAE	Stump of the LGA	31 + 59	TAE	CHA	Recovered
2/M/70	0	Surgery^a^	Active bleeding unknown	2	No chance	Diffuse bleeding	DIC; death in 24 h
10/M/67	16	Surgery^a^	The hilus of the spleen and retroperitoneal space	16	No chance	Diffuse bleeding	DIC; death in 24 h
27/M/57	23	TAE	CHA pseudoaneurysm	23	No chance	Diffuse bleeding	DIC; death in 24 h

TAE can be repeatedly utilized in patients who have re-bleeding. Half of the re-bleeding patients (5/10) were recovered after repeated TAE

*CHA* common hepatic artery, *SPA* splenic artery, *LGA* left gastric artery, *LHA* left hepatic artery, *PHA* proper hepatic artery, *POD* postoperative day

^a^In these patients, the bleeding artery was not definitely found. Active bleeding was controlled by gauze packing

## Discussion

Massive bleeding after radical gastrectomy is a less frequent complication, with the incidence ranging from 0.6 to 3.3 % in most series.^5^,^23^,^24^ However, it is a highly intractable and lethal morbidity without standardized management. Severe postoperative hemorrhage not only increases mortality rate and prolongs hospital stay but also leads to an overall poor 5-year survival rate in patients with gastric cancer.^25^,^26^ Abdominal arterial bleeding represents the most severe hemorrhage manifesting either intraluminal or extraluminal or as both, after radical gastrectomy. In the present study, we retrospectively investigated 1875 consecutive patients receiving gastrectomy with extended (D2 or D2 plus) lymphadenectomy in a single institute. The overall postoperative arterial hemorrhage rate was 1.92 %, with a relatively high mortality rate of 33.3 %.

In the early postoperative period, the main cause of bleeding is technical failure,^16^,^20^ while in the late bleeding period, the complication-associated erosion of the artery and potential arterial wall injury during lymph node dissection would be the two main interactional reasons.^14^,^15^,^18^ In our series, 80 % of late massive arterial bleeding patients (24/30) had complications of an intra-abdominal infection caused by anastomotic leakage or pancreatic fistula before bleeding. Infection, leakage, and massive arterial bleeding should be considered as the triad of lethal complication following radical gastrectomy.

**Fig. 4:**
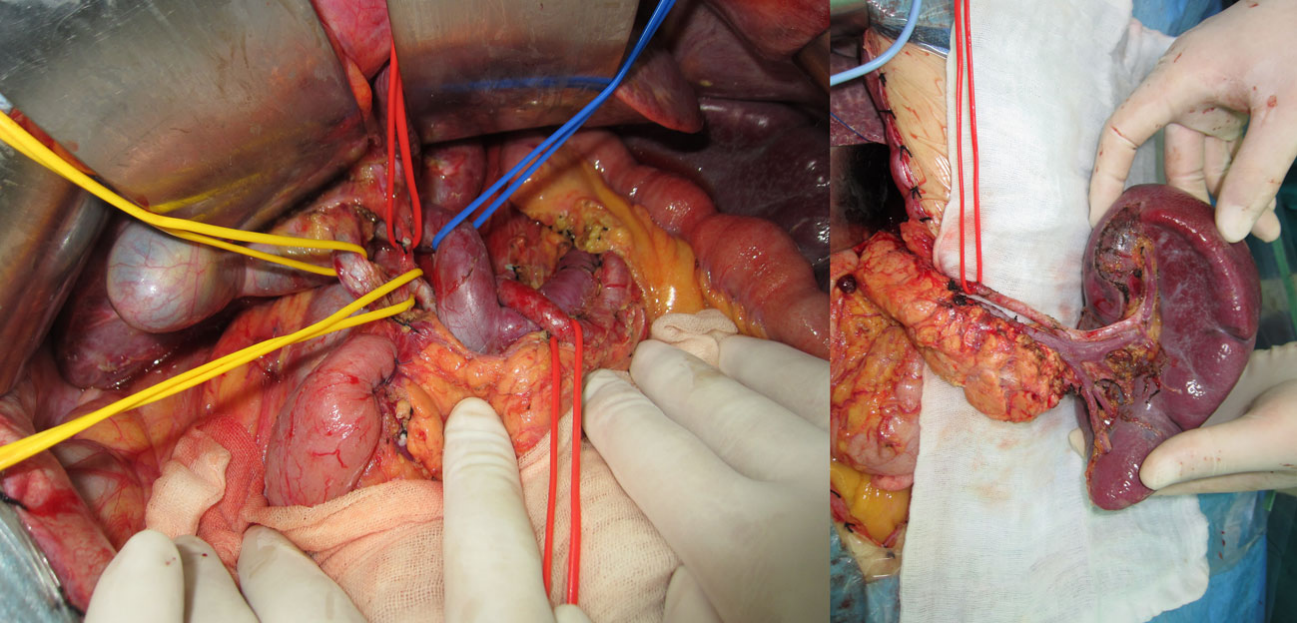
Completion of a total gastrectomy with D2 plus lymphadenectomy for gastric cancer. For patients with advanced gastric cancer, an extensive vascular skeletonization could totally remove tissues within the vascular sheath, such as neuro-lymphatic layer, lymph nodes, and interstitial fatty tissues, and achieve a radical lymphadenectomy. The *left picture* shows an anatomic variation that the portal vein went across *above* the common hepatic artery. The *right picture* shows the completion of lymphadenectomy of the splenic hilum

In the present series, we found that the two most common bleeding arteries—common hepatic artery and splenic artery, were accorded with the arteries which were required to be carried out in D2 procedures.^22^ From our experience, potential arterial injuries can happen when the arterial skeletonization maneuvers (dissection of the arterial fibrous sheath) are used for extended lymphadenectomy (Fig. 4). Vascular skeletonization and advanced stage of the disease are suggested to be high-risk scenarios for delayed massive bleeding after radical gastrectomy.^8^ The last case in our series developed bleeding on the 90th day postoperatively. This suggests that high-risk patients should be carefully monitored, even after 2 months following surgery.

Whether anticoagulation therapy contributes to postoperative hemorrhage remained unclear in the present study. Tokioka et al. recently reported that anticoagulant therapy may increase the rate of postoperative hemorrhage in elderly patients with early gastric cancer.^23^ Perioperative antithrombotic treatment was considered as an independent risk factor of postoperative bleeding.^24^ Anticoagulation or antiplatelet therapy was not routinely practiced postoperatively in our center, except for patients with intravascular stents or preexisting thrombotic disease preoperatively. None of the patients in this series were on anticoagulation or antiplatelet therapy before bleeding. To understand the relationship between anticoagulation treatment and postoperative arterial bleeding, further studies are required.

Clinical features of all early arterial bleeding cases were similar. Sudden increase in fresh sanguineous drainage from abdominal drains was observed. Nevertheless, the late arterial bleeding cases were more complex and concealed. Symptoms such as upper abdominal pain caused by fistula, abdominal infection, or intra- or extraluminal sentinel bleeding could precede later episodes of massive bleeding. Understanding these symptoms may help raise the surgeon’s vigilance for this insidious and acute postoperative complication.^27^,^28^ Rupture of the abdominal arteries can lead not only to massive extraluminal bleeding but also to intraluminal bleeding. Local compact adhesions between anastomotic leakage and eroded arteries might turn the anastomotic site to be the only way out for the blood to flow from the ruptured arteries. This may explain why only 7 of our arterial bleeding cases presented with massive gastrointestinal bleeding.

This was a retrospective study, and all bleeding cases were confirmed by arterial angiography or open surgery. The non-arterial hemorrhage and other unclear sources of hemorrhage were excluded. However, in clinical practice, sometimes it is difficult to distinguish between venous and arterial bleedings. In cases that are hard to differentiate, we would carry out active resuscitation, followed by close observation and preparation for angiography.

It is worth mentioning that in late arterial bleeding cases, we found that 70 % (21/30) of patients had at least one instance of sentinel bleeding before massive hemorrhage occurred. Schafer et al. mentioned that sentinel bleeding can be a predictor of late bleeding.^29^ Unfortunately, sentinel bleeding may be an underestimated sign of late bleeding. For those high-risk patients who received arterial skeletonization in the advanced stage following radical gastrectomy, especially with complications like abdominal infection, a delayed transient or minor intra- or extraluminal bleeding should be considered as sentinel bleeding and it could precede a massive arterial hemorrhage. Given the potentially fatal outcome, any episode of sentinel bleeding must be promptly attended with immediate diagnostics and treatments. Imaging studies and angiographies should be more actively performed to detect underlying bleeding source, such as a newly formed pseudoaneurysm.

Early postoperative arterial bleeding can be treated effectively by immediate re-laparotomy with an acceptable outcome.^7^,^14^ However, re-operation is often difficult in the late phase as the bleeding site is difficult to find due to adhesions, inflammatory reactions, and friability of postoperative tissues.^10^,^30^ The high mortality can be caused not only by massive bleeding but also by the injuries from prolonged procedures done for hemostasis. As there is an increased risk of death by the vicious cycle of hypothermia, coagulopathy, and acidosis, also known as the triad of death,^31^,^32^ those cases of arterial bleeding require a damage control strategy,^33^ such as abbreviated surgery or minimally invasive TAE. As compared to surgery, TAE is a less invasive approach, irrespective of tissue adhesions and friability caused by primary surgery. From our series, although TAE had a higher re-bleeding rate compared to re-laparotomy, it achieved a significantly lower mortality and shorter bleeding control time. In addition, TAE can be repeatedly utilized in patients with re-bleeding as observed in 5 of the 10 re-bleeding patients who recovered after repeated TAE.

Admittedly, angiographic interventions could only be performed in patients with relatively stable hemodynamic status following successful fluid resuscitation and blood transfusion. In case of a life-threatening situation, i.e., continuing hemodynamic deterioration or collapse, a bedside decision of re-operation should be made. From our study, angiography and TAE should be the preferred choice of first-line treatment for late bleeding patients, if clinical conditions permit. Even if arterial embolization is not successful, visceral angiography provides useful surgical information for an upcoming rescue re-laparotomy. Both embolization and surgery play an important role in the management of bleeding after gastric cancer surgery.^20^,^30^ However, the limitations of angiography and TAE in cases of diffuse venous bleeding or intermittent bleeding should be highlighted. When embolization fails or on a negative angiographic finding, surgery is the only salvage option. Thus, a hybrid operational theater and multidisciplinary team including gastroenterologists, interventional radiologists, anesthesiologists, and surgeons are necessary for these patients.

**Fig. 5:**
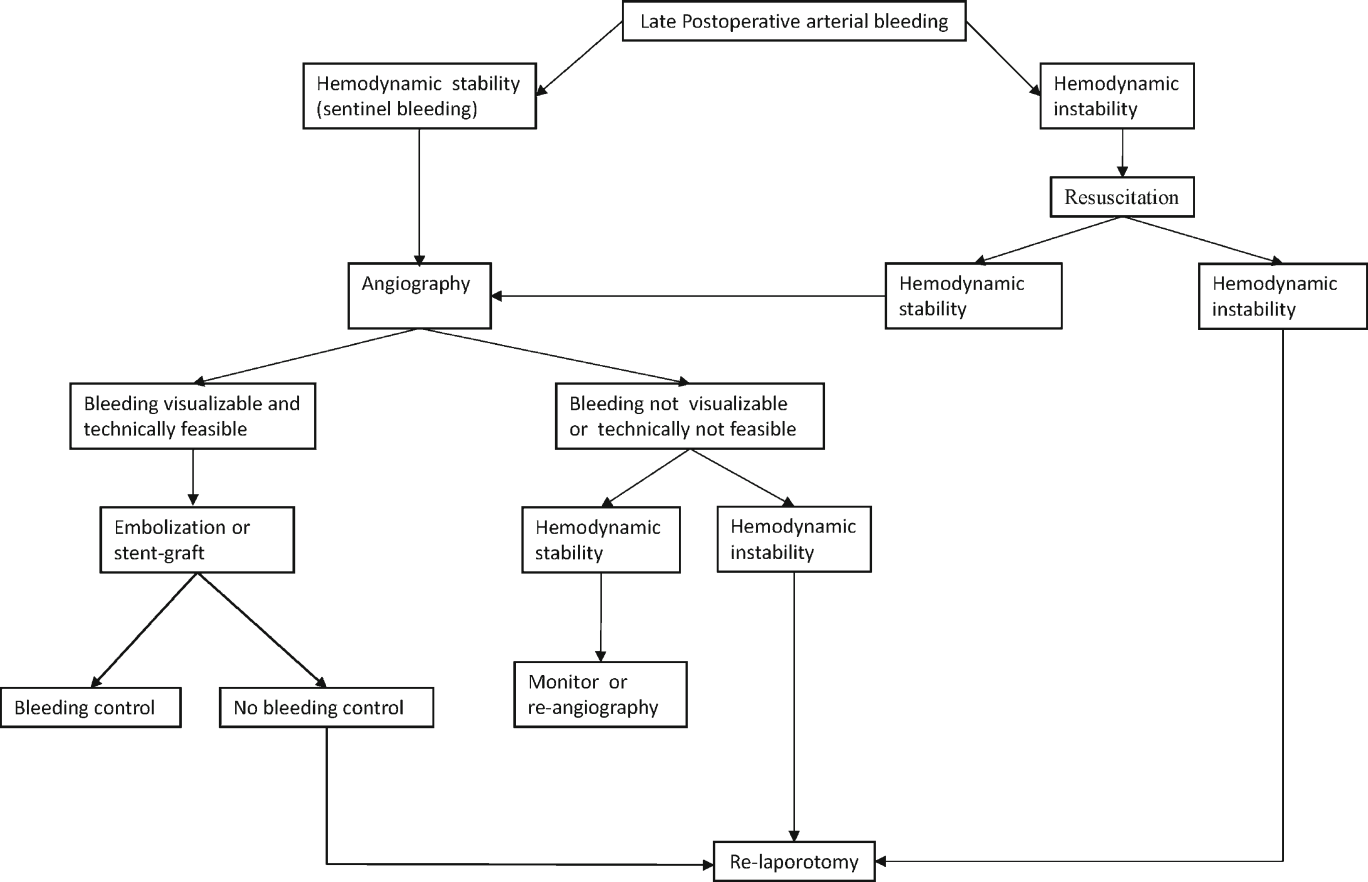
Suggested algorithm for managing late arterial bleeding following radical gastrectomy

Based on a 15-year institutional experience, we seek and suggest a diagnostic and therapeutic algorithm for late postoperative bleeding that may help to allocate patients to a customized therapy (Fig. 5).

## Conclusion

In conclusion, postoperative abdominal arterial bleeding is less common, yet lethal in patients following radical gastrectomy with extended lymph node dissection. Arterial bleeding tends to occur in the later phase after surgery with associated complications like abdominal infection and fistula. Angiographic arterial embolization is less invasive and provides better outcome for patients with late postoperative massive hemorrhage and should be considered as the choice of management when possible.

## Electronic supplementary material

ESM 1 (DOCX 27 kb)
