# Supplementary material for: Diagnosis and Treatment of Abdominal Arterial Bleeding After Radical Gastrectomy: a Retrospective Analysis of 1875 Consecutive Resections for Gastric Cancer
Source: J Gastrointest Surg. 2015 Dec 14;20:510–20. doi: 10.1007/s11605-015-3049-z (PMC4752581; doi:10.1007/s11605-015-3049-z)
Supplement: Supplementary file 1 — (DOCX 27 kb) [file 11605_2015_3049_MOESM1_ESM.docx]

**Supplemental Table. Detail information of the 36 arterial bleeding cases.**

| Patient No./sex/age, y | IAI before bleeding | Sentinel bleedings | Onset * | Treatment | Time^#^, h | Bleeding Site Assessed Intraoperatively | ICU Need | Outcome |
| --- | --- | --- | --- | --- | --- | --- | --- | --- |
| 1/M/55 | No | No | 0 | Surgery | 2 | active bleeding from the body of pancreas | No | Recovered |
| 2/M/70 | No | No | 0 | Surgery | 4 | massive active bleeding (unknown) | Yes | DIC; Death in 24-h |
| 3/M/55 | No | No | 0 | Surgery | 1.6 | artery from splenic flexure of the colon | Yes | Recovered |
| 4/M/65 | No | No | 0 | Surgery | 6 | mesocolon surface | No | Recovered |
| 5/F/64 | No | No | 0 | Surgery | 2 | active bleeding from the tail of pancreas | Yes | Recovered |
| 6/M/73 | No | No | 0 | Surgery | 2 | active bleeding from the body of pancreas | Yes | Recovered |
| 7/M/43 | No | Yes | 4 | DSA (negative finding) | ** | massive active bleeding (unknown) | Death | Death in 24-h |
| 8/F/77 | IAI | Yes | 17 | Surgery | 3.5 | CHA | Yes | MODS; death in 24-h |
| 9/M/73 | IAI+AL+DSL | No | 13 | Surgery | 4.1 | the hilus of spleen | Yes | MODS; death |
| 10/M/67 | IAI | Yes | 16 | Surgery | 6 | the hilus of spleen and retroperitoneal space | Yes | DIC; death in 24-h |
| 11/M/68 | No | No | 4 | Surgery | 4 | branch from middle colic artery | Yes | Recovered |
| 12/F/57 | IAI | Yes | 17 | Surgery | 6 | CHA | Yes | MODS; death |
| 13/M/56 | No | Yes | 31 | Surgery | 6 | SPA | Yes | Recovered |
| 14/F/42 | IAI | Yes | 15 | Surgery | 4.3 | CHA | Yes | Recovered |
| 15/M/56 | IAI+PL | No | 14 | Surgery | 5 | PHA | No | Recovered |
| 16/M/58 | No | Yes | 6 | Surgery | 7 | tail of pancreas | Yes | Recovered |
| 17/M/54 | No | No | 40 | Surgery | 6 | posterior wall of descending duodenum | Yes | MODS; death |
| 18/M/57 | IAI | Yes | 20 | Surgery | 4 | CHA | Yes | MODS; death |
| 19/M/64 | IAI | Yes | 11 | Surgery | 5 | SPA | Yes | MODS; death in 24-h |
| 20/M/65 | IAI | Yes | 9 | Surgery | 5 | CHA | Yes | MODS; death |
| 21/M/58 | IAI | No | 27 | Surgery | 3 | CHA | Death | Death during surgery |
| 22/F/69 | IAI | Yes | 10 | Surgery | 4 | upper pole of spleen | No | Recovered |
| 23/M/60 | IAI + AL | Yes | 5 | Surgery | 2 | marginal artery of transverse colon | Yes | Recovered |
| 24/M/65 | IAI + AL | Yes | 36 | TAE | 2 | LHA | No | Recovered |
| 25/M/67 | IAI+PF | Yes | 31 | TAE | 4 | accessory hepatic artery originated from SMA | No | Recovered |
| 26/M/55 | IAI+AL | Yes | 34 | TAE | 1.3 | SPA pseudoaneurysm | No | Recovered |
| 27/M/57 | IAI+PF | Yes | 23 | TAE | 3.2 | CHA pseudoaneurysm | Yes | DIC; Death in 24-h |
| 28/M/48 | IAI | Yes | 24 | TAE | 2 | CHA | Yes | Recovered |
| 29/M/53 | IAI | No | 20 | TAE | 1.5 | SPA pseudoaneurysm | Yes | Recovered |
| 30/M/54 | No | Yes | 37 | Stent graft | 1 | CHA pseudoaneurysm | Yes | Recovered |
| 31/M/58 | No | No | 90 | TAE | 1 | Gastroduodenal artery | Yes | Recovered |
| 32/M/65 | IAI + PF + AL | Yes | 26 | TAE | 3.5 | stump of LGA | No | Recovered |
| 33/M/57 | IAI+PF+AL | Yes | 30 | TAE | 2 | CHA pseudoaneurysm | Yes | Recovered |
| 34/M/62 | IAI+AL | Yes | 34 | TAE | 1.6 | SPA pseudoaneurysm | No | Recovered |
| 35/M/49 | IAI+DSL | No | 15 | TAE | 3 | pancreaticoduodenal artery | No | Recovered |
| 36/M/60 | IAI | Yes | 18 | TAE | 4 | SPA pseudoaneurysm | Yes | Recovered |

Abbreviation: DSL, Duodenal stump leakage; CHA, common hepatic artery; SPA, splenic artery; LGA, left gastric artery; PHA, proper hepatic artery; LHA, left hepatic artery; SMA, superior mesenteric artery;

**^*^** postoperative day (day);

**^#^** operating room/procedure time for hemostasis.

******This patient didn’t have chance get further surgical or TAE intervention due to acute hemodynamic collapse one hour after negative finding by angiography.
